# Possible Implication of Local Immune Response in Darier's Disease: An Immunohistochemical Characterization of Lesional Inflammatory Infiltrate

**DOI:** 10.1155/2010/350304

**Published:** 2010-06-30

**Authors:** Clelia Miracco, Francesco Pietronudo, Vasileios Mourmouras, Michele Pellegrino, Monica Onorati, Maria Grazia Mastrogiulio, Luca Cantarini, Pietro Luzi

**Affiliations:** ^1^Section of Pathological Anatomy, Department of Human Pathology and Oncology, University of Siena, Policlinico Santa Maria alle Scotte, Via delle Scotte, 6, 53100 Siena, Italy; ^2^Section of Dermatology, Department of Clinical Medicine and Immunological Science, University of Siena, 53100 Siena, Italy; ^3^Unit of Rheumatology, Interdepartmental Research Center of Systemic Autoimmune and Autoinflammatory Diseases, University of Siena, Policlinico Le Scotte, 53100 Siena, Italy; ^4^Department of Odontostomatological and Ophthalmological Science, University of Siena, 53100 Siena, Italy

## Abstract

Cell-mediated immunity is considered to be normal in Darier's Disease (DD), an inherited skin disorder complicated by skin infections. To date, there are no investigations on the local inflammatory infiltrate in DD skin lesions. In this immunohistochemical study we characterized and quantified it, making comparisons with two other inflammatory skin disorders, that is, pemphigus vulgaris (PV) and lichen ruber planus (LRP), and with the normal skin (NSk). We found a significant (*P* < .05) decrease of CD1a+ Langerhans cells (LCs) in DD, compared to PV, LRP, and NSk, and of CD123+ plasmacytoid dendritic cells (pDCs), compared to PV and LRP. We hypothesize that the genetic damage of keratinocytes might result in a loss of some subsets of dendritic cells and, consequently, in an impaired local immune response, which might worsen the infections that inevitably occur in this disease.

## 1. Introduction

Darier's disease (DD) is an infrequent autosomal dominantly inherited skin disorder caused by mutations in the ATP2A2 gene, which encodes a calcium pump highly expressed in epidermal keratinocytes [1]. DD is histologically characterized by dyskeratosis and acantholysis, due to an altered keratinization and a loss of adhesion between epidermal keratinocytes. This leads to the development of keratotic papular lesions and plaques in the seborrheic areas of the head, neck, and trunk; hypertrophic and bullous lesions, as well as mucosal involvement are more rare clinical manifestations of the disease. Disfiguring, malodorous lesions covering most of the body may also occur and fatal cases have been reported [2]. The disease runs a chronic course; topical (emollients, corticosteroids, retinoids, 5-fluorouracil), physical (excision, electrodessication, dermabrasion, ablative laser, photodynamic therapy), as well systemic (oral antibiotics, retinoids) therapies are among the treatment options, which, however, are often unsuccessful; some relief may be obtained with systemic retinoids.

 The cell-mediated immunity of DD patients was usually found to be normal [3]. There is, however, a predisposition to bacterial, fungal and viral infections, and defects in cell-mediated immunity have been described, although in a few reports [3]. It is generally believed that infections in DD occur due to the compromised skin integrity, leading to skin denudation and superimposed infections [3]. To our knowledge, there are no studies that characterized cells involved in local cutaneous immune response in DD patients.

 We herein performed an immunohistochemical analysis and quantified the inflammatory cell infiltrate in skin biopsies of DD patients, aiming at investigating a possible pathogenic involvement of the local immune response in the disease. For comparison, lesional skin of patients affected by pemphigus vulgaris (PV), as well as cutaneous lichen ruber planus (LRP) was also examined.

## 2. Subjects and Methods

We analyzed 16 skin biopsies of patients with Darier's disease (DD), ranging in age from 25 to 50 years (females outnumbered males from 9 to 7). Nine patients had multiple affected relatives, while 7 individuals represented sporadic cases. Six patients were at the first DD attack, while 10 presented multiple attacks. Skin biopsies were performed in recently-appeared, uncomplicated lesions, on grouped keratotic papules of the abdomen and trunk. There was not any personal nor family history of autoimmune disease. 

For comparison, 14 patients with pemphigus vulgaris (PV), ageing 30–55 years, and 10 with lichen ruber planus (LRP), ageing 32–51 years, were also included in the study. The diagnosis was previously established by clinical and histopathological examination, and, in PV group, also by immunofluorescence studies. As negative control, we examined normal skin (NSk) excised from the abdomen of 12 healthy subjects who underwent aesthetic surgery. Informed consent was obtained from all patients, and the study, performed according to local ethical guidelines, was approved by the local ethical committee.

## 3. Immunohistochemistry

4-*μ*m-thick sections, cut from formalin-fixed, paraffin-embedded skin biopsies, were investigated immunohistochemically.

In all cases, leukocyte subsets were characterized using a panel of antibodies, including antiCD3 (CD3 polyclonal antibody; Neomarkers; San Diego, Ca; dilution 1 : 1000), antiCD4 (CD4 polyclonal antibody, 4*β*12; Menarini, Florence, Italy; dilution 1 : 50), antiCD20 (CD20 monoclonal antibody, clone L26; Bio-Optica; Milan, Italy; dilution 1 : 150), antiCD8 (CD8 monoclonal antibody, CD8-144B clone; Dako; Milan; dilution 1 : 50), antigranzyme B (granzyme B monoclonal antibody, GZBO1 clone; Bio-Optica; dilution 1 : 100), antiCD25 (CD25 polyclonal antibody; Bio-Optica; dilution 1 : 50; trypsin pretreatment) antiFOXP3 (FOXP3 monoclonal antibody, Abcam; DBA, Milan, Italy; dilution 1 : 50; microwave pretreatment), antiCD1a (clone AB-5 010; BioOptica, Milan; dilution 1 : 50; EDTA pretreatment;), antiCD68 (IgG, clone PG-M1; Dako; dilution 1 : 300), and antiCD123 (6H6, eBioscience, San Diego, CA, dilution 1 : 100). Antigen retrieval was obtained either with Wcap buffer (pH 6, 98°C for 40 min), using the Ultravision Detection System antiPolyvalent HRP (LabVision, Fremont, CA, U.S.A.; Bio-Optica); or with microwave pretreatment, using the Ultravision LP Detection System AP polymer (LabVision). Sections were then incubated with the antibodies for 60°C min at room temperature. For staining development, we used either diaminobenzidine or fuchsin (DAB; Dako), as chromogens. We also performed a double staining with antibodies against CD25 and FOXP3 to identify the CD4+CD25+FOXP3+ subset of regulatory T cells (Tregs), as previously described [4], and against human CD68 and CD123 to identify the CD123+ pDCs. Treg and pDC nuclei were stained in red and their cytoplasm in brown. Negative controls were obtained by replacing the specific antibody with nonimmune serum immunoglobulins at the same concentration as the primary antibody. Sections were then counterstained with Harris haematoxylin, dehydrated in alcohol, cleared in xylene and coverslipped.

## 4. Quantification of the Inflammatory Infiltrate

In all samples, immunohistochemical staining was quantified visually by two independent observers (C.M. and G.M.) in at least 5 randomly chosen high power fields (HPF) (×40 objective and ×10 eyepiece; 0.16 mm^2^ per 1 HPF). CD4+, CD8+, granzyme B+, CD20+, CD25+/FOXP3+, and CD68+CD123+ cells were counted and recorded as a percentage of the total inflammatory infiltrate. CD1a+ LCs were counted separately in the epidermis and dermis, and given as mean number/1 HPF. Mean values + SD of immunostained cells were statistically compared among DD, PV, LRP and NSk by the nonparametric Kruskal-Wallis test. The significance level was chosen at *P* < .05.

## 5. Results

**Figure 1:**
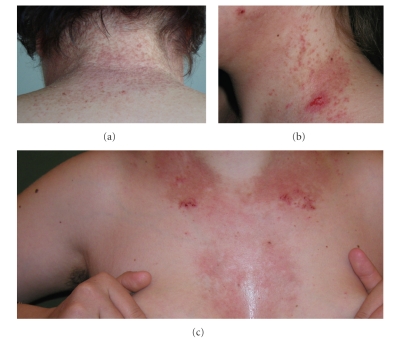
Clinical manifestations of the disease, with keratotic papules ((a), (b)), erythematous plaques, and crusted lesions ((b), (c)).

Clinics was typical of DD (Figure 1). At histology, DD cases showed suprabasilar acantholysis, dyskeratosis, with overlying columns of parakeratosis, and a usually mild, mostly lymphocytic, inflammatory infiltrate.

Blisters, due to a suprabasilar detachment, and a mild inflammatory infiltrate, with admixed lymphocytes and eosinophils, characterized PV lesions. 

 Hyperkeratosis, hypergranulosis, acanthosis and a dense, predominantly lymphocytic, band-like, subepidermal inflammatory infiltrate was observed in LRP samples. 

**Figure 2:**
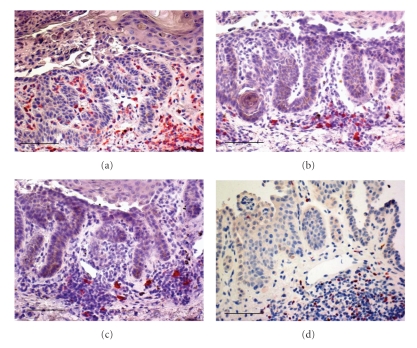
The inflammatory infiltrate in Darier's disease is mostly constituted by CD4+ T lymphocytes (a), with less numerous CD8+ T (b), and a few CD20+ B (c) lymphocytes. Redstained, FOXP3+ nuclei of Tregs are shown in (d) Immunohistochemistry; original magnification, ×200; Scale bar = 75 *μ*m; Chromogen: fuchsin.

 In all three diseases CD3+ T lymphocytes outnumbered CD20+ B lymphocytes (Figure 2). 

In DD, CD4+ T lymphocytes constituted the large majority of inflammatory cells, with a minor component of CD8+ T lymphocytes (Figure 2); very few granzyme B+ lymphocytes were observed. Viceversa, in PV and in LRP, high numbers of T cells were of the CD8 phenotype, and many granzyme B+ positive cells were observable. Lymphocytes were rarely observed in the NSk.

**Figure 3:**
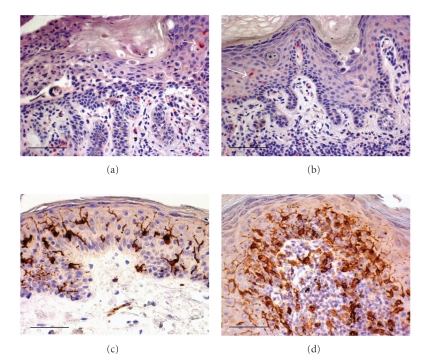
Few CD1a+ LCs (an intraepidermal CD1a+ LC is indicated by the arrow), small in size, and without evident dendrites, in two cases of Darier's disease ((a), (b)). Normal skin showing more numerous, larger CD1a+ LCs, with long dendrites (c). An increased number of both epidermal and dermal CD1a+ LCs in a case of lichen ruber planus (d). Immunohistochemistry. Original magnification, ×200; Scale bar = 75 *μ*m; Chromogen: fuchsin ((a), (b)); diaminobenzidine ((c), (d)).

 In DD the epidermal, as well as the dermal CD1a+ LCs were noticeably decreased in lesional skin when compared with PV and LRP lesions, and NSk (Figure 3). LCs were also smaller in size and with fewer and shorter dendrites, when compared to NSk. In the perilesional DD skin, when documentable in the biopsy, CD1a+ LCs reappeared, although less numerous than those in NSk. In PV the CD1a+ LCs appeared as isolated cells or in small clusters in the epidermis as well as in the dermis; more numerous than those in DD, they, however, appeared decreased when compared to LRP and NSk and often reduced in size. In LRP, both epidermal and dermal CD1a+ LCs were, instead, detectable in high numbers, with evident, long dendrites, and often grouped together.

**Figure 4:**
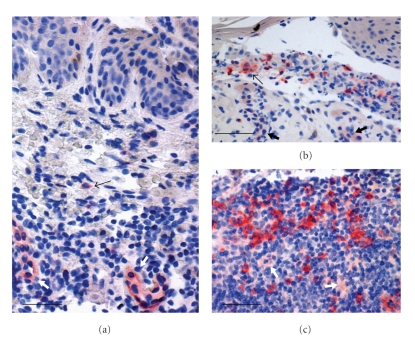
A few CD123+ pDCs, as the one indicated by the thin arrow, in the inflammatory infiltrate of a Darier's disease case (a). In pemphigus vulgaris, CD123+ pDCs are more numerous, usually found in the dermis and/or inside the bulla ((b), thin arrow). A lichen ruber planus skin sample showing a high number of single or grouped CD123+ pDCs in the dermal inflammatory infiltrate (c). The endothelium of vessels ((a), (b), (c), thick arrows) is also weakly stained by the antiCD123 antibody. Immunohistochemistry; original magnification, ×200. Scale bar: A = 35 *μ*m; B, C = 45 *μ*m; Chromogen: fuchsin.

 Dermal plasmacytoid dendritic cells (pDCs) were identifiable based on both their plasmacytoid morphology and positivity for CD123. CD123 was also expressed by endothelial cells, and therefore they served as an internal positive control for the antiCD123 antibody (Figure 4). CD123+ pDCs were observed, at low percentages, usually single or in groups of two, in the dermis in DD; at higher percentages, preferentially in the dermis and inside the bullae, but also in the epidermis in PV; in greater numbers, single and, more often, in coalescent groups of cells, in the dermis in LRP. CD123+ DCs were virtually absent in the NSk.

## 6. Statistics

Quantitative descriptive data of results and *P* significance are given in Table 1. CD3 and CD20 did not significantly differ among the three groups. CD4+ T lymphocyte percentages significantly differ from CD8+ percentages in each group of lesions, CD4+ percentages being higher than CD8+ in DD, and lower in PV and LRP. CD8+ T lymphocytes were found at high percentages in both PV and LRP. Percentages of CD4+, and CD8+ T lymphocytes did not statistically differ between PV and LRP. In DD, instead, CD8+ and CD4+ lymphocytes were, respectively, significantly lower and significantly higher than those in PV and LRP. Granzyme B lymphocyte percentages were significantly lower in DD than those in PV and LRP; and in PV than those in LRP.

 Mean percentages of CD25+FOXP3+ Tregs were significantly higher in DD than those in PV and LRP; they did not differ between DD and LRP.

 In DD, epidermal and dermal CD1a+ LC mean number, as well as CD123+ DC percentage were significantly lower than those in PV, LRP and in the NSk. In PV, epidermal and dermal Cd1a+ LC mean numbers were significantly lower than those in LRP. In PV epidermal CD1a+ DC mean number was also significantly lower than those in NSk.

CD123+ DC mean percentages were significantly lower in DD than those in PV and LRP; they were also significantly lower in PV than those in LRP.

 Comparison with the NSk was limited to CD1a+ LCs, due to the virtual absence of lymphocytes and plasmacytoid cells in controls.

## 7. Discussion

A genetic basis corroborate Darier's disease (DD), an autosomal dominant genodermatosis, with sporadic cases reported, in which an aberration localized to the 12q23-24.1 region of chromosome 12 has recently been discovered, leading to a defect in a keratinocyte calcium pump, which, in turn, results in a loss of keratinocyte adhesion and consequent acantholysis, typical of the disease [1]. Autoimmunity is, instead, the main cause of pemphigus vulgaris (PV) and lichen ruber planus (LRP), in which altered immune response, as well as skin inflammatory cell composition, functionality and immune profile have been well documented [5–13]. DD and PV are both characterized by epidermal suprabasilar acantholysis, due to loss of adhesion between keratinocytes in the former, and caused by autoantibodies in the latter. Despite an ascertained predisposition to bacterial, fungal and viral infections, no specific immunological abnormality has been discovered in DD [3]. Infective complications are ascribed only to superimposition of secondary infections on the denuded skin areas [3]. 

 The observation that a usually mild inflammatory infiltrate is, however, detectable also in of recent onset, uncomplicated DD skin lesions, prompted us to characterize it by immunohistochemistry, in order to investigate cell composition of the local immune response; we also made comparison with inflammatory infiltrate of PV and LRP skin lesions. In the latter conditions, our results are in line with the literature [5–13]. To our knowledge, there are no investigations, to date, that characterized the inflammatory infiltrate in DD. In all DD skin lesions we found that it was mostly represented by CD4+ T lymphocytes, with a minor component of CD8+ T lymphocytes, and a negligible percentage of CD20+ B lymphocytes. The most striking finding in all DD cases, in our experience, was a loss of epidermal CD1a+ LCs in the affected acantholytic and dyskeratotic skin areas. They reappeared abruptly in the neighbouring, apparently normal epidermis, although at lower number, when compared to the normal skin (NSk). We also observed low percentages of a subset of dermal dendritic cells, the so-called plasmacytoid DCs, identifiable by CD123 positivity [14]. It has been demonstrated that keratinocyte signals induce LC proliferation [15]; keratinocytes secrete cytokines that promote LC residence and regulate their immune functions [16], and it has been suggested that cytokeratin differentiation provide a microenvironment that determines the density of Langerhans cells [17]. The profound defect of keratinocyte differentiation in DD, could, therefore, result in epidermal LC loss in the affected epidermis. Basically, epidermal LC functions are largely unknown [15], however, although debated, their cooperation in generating specific apten-induced effector T cells and stimulating T-cell immunity was demonstrated in some studies [18–21]. LCs were found capable of promoting and regulating T cell–mediated immune response [22–24], and were potent activators of CD8+ T cells in the skin [25]. Epidermal LC reduction or loss might contribute to a CD8-mediated effector local immune response impairment, and, consequently, to a poor response to infective agents in DD. Furthermore, since keratinocytes seem to function as accessory cells competent to prime naive skin-reactive T cells [26], their defect in DD, might directly determine an impaired CD8-mediated effector immune response. 

 It is known that UV irradiation exacerbates DD cutaneous lesions [2, 3]: this might partly be due to a direct modulation of keratinocytes by UV to produce proinflammatory cytokines such as interleukin (IL)-1, IL-6, IL-8 and tumour necrosis factor (TNF)-A [27], leading to skin inflammation. Based on the hypothesis of a haploinsufficiency in DD, it was additionally hypothesized a worsening of cutaneous lesions due to a direct downregulation of the intact allele of the ATP2A2 gene by cytokines secreted by UV-irradiated keratinocytes, leading to an altered intracellular distribution of calcium, and, consequently, to a further loss of epidermal cell adhesion, which is modulated by the cellular calcium level [1]. On the other hand, it has been ascertained that UV induces immunosuppression [28, 29], probably through more than one mechanisms and targeting various cell types, including keratinocytes, antigen presenting cells, such as epidermal Langerhans cells (LCs), and T lymphocytes. UV halts antigen presentation functions, and, furthermore, expands T cell population with regulatory properties on effector immune cells [29].

 Although, at least in experimental studies on mice, a dermal subset of LC rather than epidermal LC was critical in the UV-induced immunosuppression [30], many studies demonstrated numerical, morphological, phenotypical and functional abnormalities of epidermal LCs following UV exposure [29–33]. UV seems to affect LCs directly or indirectly through suppressive cytokines [29]. In DD skin, UV might, therefore, further on reduce LC number, that we observed both in epidermis and dermis, contributing to a local immunosuppression. 

 In our study, we also found low percentages of dermal pDCs, as decorated by CD123 antibody, which labels interleukin-3 receptor alpha chain protein, that is expressed at high levels on the surface of pDCs, allowing for their identification [14]. 

 The DC system consists of two main subsets: myeloid DCs (mDCs) and plasmacytoid DCs (pDCs) [25], and at least five distinct DC subsets have been discovered in the skin [34]. Epidermal CD1a+ LCs, dermal CD1a+ LCs and CD14+ DCs are the most known mDCs in the skin, dermal CD1a+ DCs bearing intermediate characteristics between the first two subsets [25]. It was hypothesized that the humoral and the cellular arms of adaptive immunity are preferentially regulated by CD14+ dermal DCs, and LCs, respectively [25]. An emerging role of the plasmacytoid subset of DCs has recently been evidenced in several skin diseases, in which they have been found in high numbers [35, 36]. In NSk, pDCs are low or absent [14], and they are not altered by UVB [37]. CD123+ pDCs are capable of producing type I interferon, and there is evidence that pDCs are the main source of this cytokine in skin inflammation [36]. CD123+ pDCs have in fact been found involved in antiviral immunity and in several autoimmune disorders [25, 35–37]. Interferon-alpha is able to induce the production of chemokines, which, in turn, recruite CD8+ T cells and enhance the expression of cytotoxic mediators by NK cells and cytotoxic lymphocytes [38, 39]. A low number of pDCs, as we observed in DD, might result in a low production of interferon, and interferon-inducible chemokines, leading to an explosion of infectious agents, because of inadequate cytotoxic local immune response. We cannot, however, assess whether the altered number of CD123+ pDCs that we registered might be ascribed to the complex interactions with other inflammatory cells, including CD1a+ LCs, or to other factors. 

 In our experience, also in PV, we found impaired numbers of both CD1a+ LCs and CD123+ pDCs. We, in fact, found that in PV, CD1a+ LCs were lower than those in NSk and LRP, and that CD123+ pDCs were lower than those in LRP, although both DC subsets were significantly higher than those in DD. PV is also characterized by acantholysis, overwhelmingly due to autoimmune mechanisms; it might be hypothesized an additional contribute of damaged keratinocytes to the decrease of some DC subsets. 

 We, furthermore, observed a different pattern of CD123+ DC distribution (dermal in DD and LRP, intraepidermal and dermal in PV), which is supposed to have pathogenic implications in distinct diseases [40]. 

 In line with other studies [41], we registered significantly lower numbers of CD25+FOXP3+ regulatory T cells (Treg) in PV and LRP, whereas, in DD, their percentages were within the range normally reported in inflammatory infiltrates. 

 Tregs are a phenotypically heterogeneous population of lymphocytes which are critical for the regulatory mechanisms of immune system, being responsible for the induction and maintenance of immune tolerance. Their numbers, and, overall, their suppressive functions were found reduced in autoimmune as well as in allergic diseases [8, 40, 41].

 FOXP3 is considered the best marker for the most important among Treg subsets, the CD4+CD25+FOXP3+ cells, that constitute approximately 5–10% of CD4 T cells in man [41]. The scenario of well-represented CD4+ T infiltrates, with CD25+FOXP3+ within the normal range, in front of a low number of effector cells in DD skin, although the presence of inflammatory cells itself does not necessary mirror their functionality, suggest a defect of effector rather than an enhancement of regulatory cells being effective in DD. Dendritic cell numerical impairment seems the first Cause of a defective local immune response.

## 8. Conclusion

In conclusion, our present study provides direct evidence for a numerical impairment of epidermal CD1a+ LCs and dermal CD123+ pDCs in skin lesions of DD, which may be pathogenetically determinant for the development of the inflammatory reactions that complicate the disease. To our knowledge, this investigation represents the first description of an altered composition of the cellular inflammatory infiltrate in this genetical disorder, suggesting that an impaired local immune response may contribute to the progression of the disease. The dramatic loss and/or absence of some skin DC subsets, such those that we observed, might drive the pathogenic events leading to DD skin complications, suggesting novel therapeutic approaches that could open new horizons for the treatment of this notoriously poorly responsive disease and for a better management of patients. Further functional studies, as well as expanding investigation on other cells and cytokine mediators of immune response and inflammation are, of course, mandatory to better elucidate the impact of epidermal LC and dermal CD123+ DC loss in DD lesional microenviroment.

## Figures and Tables

**Table 1a:** (a) Immunohistochemically-characterized inflammatory infiltrate in Darier's disease (DD), pemphigus vulgaris (PV), and lichen ruber planus (LRP). Mean percentages (%) +SD and range of values in brackets are reported. *P* significance is reported at the bottom.

	DD	PV	LRP
	% ±SD (range)	% ±SD (range)	% ±SD (range)
CD20+	11.1 ± 4.4 (1–20)	12.6 ± 3 (8–20)	13.2 ± 3.8 (5–25)
CD3	90 (80–95)	90 (85–95)	85 (80–95)
CD4+	70 ± 6.2 (50–80)	47.8 ± 8.6 (30–60)	49.7 ± 5.8 (35–60)
CD8+	20 ± 5.4 (10–30)	40.6 ± 8.9 (25–55)	40 ± 5.7 (30–50)
Granzyme B+	3.3 ± 2.7 (0.4–0.8)	16 ± 4.8 (10–30)	18.3 ± 4.7 (10–30)
CD25+FOXP3+ Tregs	26 ± 8.5 (8–40)	11.5 ± 4.7 (1–20)	12.7 ± 4.7 (8–35)
CD68+CD123+ pDC	0.6 ± 0.3 (0.2–1)	3.1 ± 1.1 (0.9–5)	9.1 ± 1.7 (6–13)

**Table 1b:** (b) Mean number (N) ± SD and range of values in brackets of epidermal (e) and dermal (d) CD1a+ LCs/1 HPF, in DD, PV, LRP, and in the normal skin (NSk). *P* significance is reported at the bottom.

	DD	PV	LRP	NSk
	N ± SD (range)	N ± SD (range)	N ± SD (range)	N ± SD (range)
eCD1a+ LCs	2.4 ± 2 (0–7)	5.4 ± 1.7 (3–10)	20.9 ± 3 (15–28)	9.9 ± 2.1 (7–14)
dCD1a+ LCs	3.4 ± 1.1 (2–7)	4.9 ± 1.6 (2–7)	18.5 ± 5.5 (10–26)	5.7 ± 1.4 (4–8)

*P* not significant for: CD3 and CD20, among DD, PV, and LRP groups; and for CD4+, CD25+FOXP3+ Tregs, and CD8+ in PV versus LRP.

For all the other comparisons, *P* < .05.

LCs: Langerhans cells, Tregs: regulatory T cells, and pDCs: plasmacytoid dendritic cells.
